# The Stomach Tube

**Published:** 1914-04

**Authors:** George M. Niles

**Affiliations:** Professor of Gastro-Enterology and Clinical Medicine, Atlanta Medical College, Atlanta, Ga.


### Journal-Record of Medicine

Successor to Atlanta Medical and Surgical Journal, Established 1855
and Southern Medical Record, Established 1870
**OWNED BY THE ATLANTA MEDICAL JOURNAL COMPANY**
PUBLISHED MONTHLY

*Official Organ Fulton County Medical Society, State Examining Board, Presbyterian Hospital, Atlanta, Birmingham and Atlantic Railroad Surgeons' Association, Chattahoochee Valley Medical and Surgical Association, Etc.*

EDGAR BALLENGER, M. D., Editor
BERNARD WOLFF, M. D., Supervising Editor
A. W. STIRLING, M. D., C. M., D. P. H.; J. S. HURT, B. Ph., M.D.
GEO. M. NILES, M. D., W. J. LOVE, M. D., (Ala.); Associate Editors
E. W. ALLEN, Business Manager

**COLLABORATORS**

DR. W. F. WESTMORLAND, General Surgery
F. W. McRAE, M. D., Abdominal Surgery
H. F. HARRIS, M. D., Pathology and Bacteriology
E. B. BLOCK, M. D., Diseases of the Nervous System
MICHAEL HOKE, M. D., Orthopedic Surgery
CYRUS W. STRICKLER, M. D., Legal Medicine and Medical Legislation
E. C. DAVIS, A. B., M. D., Obstetrics
E. G. JONES, A. B., M. D., Gynecology
R. T. DORSEY, JR., B. S., M. D., Medicine
L. M. GAINES, A. B., M. D., Internal Medicine
GEO. C. MIZELL, M. D., Diseases of the Stomach and Intestines
L. B. CLARKE, M. D., Pediatrics
EDGAR PAULIN, M. D., Opsonic Medicine
THEODORE TOEPEL, M. D., Mechano Therapy
R. R. DALY, M. D., Medical Society
A. W. STIRLING, M. D., Etc., Diseases of the Eye, Ear, Nose and Throat
BERNARD WOLFF, M. D., Diseases of the Skin
E. G. BALLENGER, M. D., Diseases of the Genito-Urinary Organs

VOL. LXI. ATLANTA, GA., APRIL, 1914. No. 1

### THE STOMACH TUBE

GEORGE M. NILES, M. D.
Professor of Gastro-Enterology and Clinical Medicine, Atlanta Medical College, Atlanta, Ga.

Although there were some random attempts to explore the stomach by the use of a tube before the days of Kussmaul, it was he, who in 1867 first inaugurated intelligent and systematic

WI
AT459H

methods. In 1871, Leube began also to demonstrate its use, and to these two worthies we owe much of our present-day knowledge concerning this useful diagnostic and therapeutic instrument.

Unfortunately, the prevailing attitude of the public towards the stomach tube is anything but favorable—in many instances amounting to an actual repugnance. I have known many patients, who have spent sleepless nights in awsome anticipation of the trying ordeal, and others, who would suffer for months, rather than submit to what they considered a horrible torture. So often do I hear some intelligent patient say, "Doctor, I would have been to you for aid long ago, had I not dreaded to take that awful stomach tube."

The reason for this is not hard to find. It lies in the careless, inexpert, or principally slow technic of those who have introduced the tube, and have inflicted upon patients such needless discomfort, that they have not only become themselves prejudiced, but have spread abroad the evil tidings. Candidly, I can not blame some of them, and it is the duty of the physician of this day to learn how to introduce a tube so deftly that this prejudice will be dissipated.

There are certain contraindications to the employment of the stomach tube, and these I will first make plain:

(1) Pregnancy, especially if advanced beyond the fifth month. The first few months should make no difference, and even the later months, if the patient is accustomed to the tube, and the operator is expert. If, however, the patient has not taken the tube before, the risk is grave.

Several years ago in a New York clinic I saw a tube introduced into the stomach of a Jewish woman apparently eight months pregnant. She struggled violently, and when she arose there was about half a pint of amniotic fluid in the bottom of the chair.

(2) Organic heart disease with broken compensation. To this may be added so-called cardiac neuroses, angina pectoris, real or pseudo, myocarditis, or any condition where the heart seems decidedly below par.

In some of these conditions, where the cardiac lesion does not appear to be acute, if the patient is quite phlegmatic, or if the operator is expert, risk may be taken, but the physician should realize that it is somewhat of a risk.

(3) Aneurysm of any of the large arteries. This condition absolutely contraindicates.

(4) Recent hemorrhages of any kind. This heading may include hemorrhage from the stomach, intestines, lungs, bladder, uterus, apoplexies, or hemorrhagic infractions.

A fairly good rule is to permit from ten days to two weeks to elapse, after one of these hemmorrhages, before attempting to pass the stomach tube.

(5) Advanced pulmonary tuberculosis, or advanced pulmonary emphysema, with bronchitis. The evident condition of the patient in these advanced pathologic states will generally show the inadvisability of attempting to pass a tube.

(6) Advanced arterio-sclerosis. The tube is generally contra-indicated in these cases, whether or not the blood pressure is abnormally high. Even under the most favorable psychic conditions they seldom take the tube kindly, and it is seldom wise to attempt it, especially if the blood pressure is high.

(7) Advanced cachexia from any cause. In some of these extremely enfeebled states, any manipulation entailing the slightest shock may turn the scales unfavorably, and the introduction of the tube should not be attempted, unless for very strong reasons.

(8) Evidences of erosions of the gastric mucosa, either malignant or non-malignant. In this may be included palpable carcinoma of the stomach or pylorus. accompanied by vomiting of coffee-ground material. These symptoms being present, the introduction of a tube, no matter how carefully performed, may cause serious damage. It is unwise also to explore the stomach with a tube when there are strong indications of either a single open ulcer or multiple erosions, even though they be shallow.

(9) Acute febrile conditions. All of these may not constitute a bar to the use of the tube, but the operator will find lae.

these patients very unresponsive, and, unless great benefit is likely to be accomplished, he will find that more discomfort is inflicted than good is accomplished.

(10) Those rare and peculiar conditions where upon the slightest touch of the tube against the epiglottis, the larynx spasmodically closes, and the patient becomes blue. These conditions have not been explained, but they occasionally are in evidence, and must be taken account of. This does not mean those nervous, struggling patients, who vow they are choking, when they are not, and who need only to exercise self-control. It means those, who, in "cold blood" try faithfully and intelligently to swallow the tube, but whose whole respiratory machinery automatically closes upon the slightest touch of the tube, while the face becomes livid, if the effort is kept up. These individuals simply possess an idiosyncracy against the tube, and it is useless to attempt to force it upon them.

(11) Those patients, who are violently nauseated at the merest touch of the tube upon the tongue, epiglottis or any part of the fauces, or who incline to vomit at the sight or mention of it. There are some hypersensitive individuals, who really seem in good faith unable to bear the sight, mention or touch of a stomach tube, whose very nature revolts against its use, and who cannot help this feeling. I have seen a few such "freaks," and can affirm that scarcely any diagnostic information can recompense the physician for the strenuous efforts required to extract a test meal from their frenzied stomachs.

The choise of a tube is quite important. The size for an adult should vary from 18 English or 30 French to 20 English or 32 French. Smaller than this renders the tube rather small and soft. Larger numbers are used by some, up to 24 English, and while an ordinary esophagus can accommodate them comfortably, the size is inclined to be alarming. To a fearful patient a tube always looks really larger than it is, and if he catches a glimpse of a "24," he will probably exclaim, "Great Jehosophat! Doctor, you don't expect me to swallow that hose, do you?" It is much easier to use a smaller tube, than to quiet his perturbed feelings.

For children, tubes may be used as small as 12 English or 21 French; or best 15 English or 25 French. The latter can be inserted into the esophagus of a four-year old child, and it is rarely necessary to introduce a tube in a younger patient.

The tube should be of red rubber, and fairly stiff. When lacking in a certain amount of rigidity, it is liable to coil up in the mouth instead of passing on down the esophagus, and later on, when the esophageal muscles or the cardiac opening of the stomach are resenting its onward progress, it is liable to "buckle" to the discomfort of the patient and the inconvenience of the physician. The use of a wire or any other appliance to stiffen the tube I have not found helpful.

Up to a few years ago the imported were much the best, but at present tubes can be obtained in America, fashioned and moulded to suit the most fastidious.

In regard to the number, size and character of the openings of the lower end of the tube, there is some difference in opinion, though all unite in condemning that abomination, the tube with a sharp-edged opening in the center. There is no estimating the damage done to delicate gastric mucosae by these dangerous tubes, and none such should ever be employed. Some advocate tubes with from three to eight, or even multiple openings near the end for the extraction of test meals. In my experience, however, I have obtained the best results from a tube with two openings, one about half an inch above the other and on the opposite side of the tube. These openings should be of "velvet finish," so as to inflict no injury to the mucosa, should it be drawn into the openings by force of aspiration. The end of the tube should be closed, smooth and round.

Lavage tubes should possess from two to five openings, though I consider five about the proper number. Where the tube is to be used in an empty stomach, a great number of very small openings is feasible; but generally there are remnants of food or shreds of mucus to be removed through the tube, and then the small openings are a nuisance.

As a general rule the lavage tube should be a little larger than the one for extracting a test meal. In the first place, more room is required successfully to clean the stomach, and besides, the patient has learned more about the tube and can be induced to swallow a larger one without serious objection. Judgment, though, must at all times be exercised in the size to be employed.

For lubricating the tube, plain water is by far the best. Glycerine, oils of various sorts, cream and a host of other agents have been advocated, only to be discarded. The supremacy of water admits of no argument.

Many efforts have been put forth to render insensitive the throat and fauces. There have been recommended a 3 per cent. solution of cocaine, or a 5 per cent. solution of orthoform to be sprayed over the fauces. Even a spray of ether or ethyl chloride has been attempted. Others have painted the throat and fauces with collodion, or a solution of adrenalin, or even a 5 per cent. solution of nitrate of silver. Let me deprecate the use of all these methods. None of them can take the place of skill and celerity in introducing the tube, and none or all of them can atone for the lack of these attributes on the part of the physician.

When the physician prepares to extract a test meal from a patient who has never undergone the experience, he should enter upon his task with kindness, gentleness and patience. He should spend several minutes in reassuring the patient, calming his fears, and soothing his hypersensitive nerves. The doctor should tell him that the information to be obtained is likely to prove of the utmost value in diagnosis and subsequent relief of symptoms, and that the slight inconvenience will be more than compensated by the insight afforded into obscure ills. The patient should always be interrogated as to false teeth, and if worn, they should be removed. This inquiry should never be omitted, for occasionally very young persons have them, and they are fitted in so naturally, that their presence is not suspected.

There are different methods employed to protect the patient and his clothing while a test meal is being extracted, some of them most cumbersome and fear-inspiring. Too many "protectives," rubber aprons, etc., are not advisable, in my opinion, and I have come to use two plain towels—one around the neck, and one in the lap. A plain basin may be placed in the lap, or a small porcelain basin. Too many preparations will certainly alarm the patient.

Should there be an assistant at hand, the patient need only hold the basin; otherwise he is instructed to hold the glass retainer in his left hand, and the basin in his right, the operator admonishing him to hold tightly to both, and on no account to drop them.

Up to this time the patient has not seen the tube, and he should not be permitted to see it until the instant it is to be used. If he has an opportunity to gaze on it too long, he may refuse altogether to swallow it.

Now as to the actual method of introducing this tube: I realize that the methods are legion, and that some operators get satisfactory results by means that would give no results whatever to others.

I will briefly mention some of these methods, and then give in detail the one that proved the best in my hands:

Dr. Max Einhorn stands directly in front of the patient, tells him to open his mouth, presses down the tongue with two fingers of his left hand, puts in the tube, and has it in the stomach in an incredibly short space of time. This answers in Dr. Einhorn's skilled hands, but would not in one less practised, for there is nothing to prevent the patient from rearing back and escaping from the tube, if he so desired.

Others stand either in front of the patient, or slightly to the left, and attempt to start the introduction of the tube at the proper place by the aid of sight, as the mouth is widely opened. This has proved a poor way, for here again the physician has but little control of the patient, who, with his mouth wide open and his head thrown back, is more apt to be nauseated than when his mouth is nearly closed.

The best way is to stand at the right of the patient, with the left arm of the physician lightly placed around his neck, and the left hand in front of his mouth so as to guide and control the tube. Asking the patient to slightly (not widely) open his mouth, the tube is gently but quickly inserted between the lips, and pressed back against the epiglottis. This is done by the right hand of the operator, while the tube runs between and is steadied by the two fingers of his left hand that are directly over the patient's mouth. The patient is then gently asked to swallow, and the gentle injunction is repeated several times until he makes the effort to swallow. Just as he does this, the tube will slip by the epiglottis and down the esophagus—not in the larynx, for it is almost impossible for the tube to penetrate there. Should at this instant the patient complain of choking, admonish him to swallow again, and when he finds he can do so, he will have no more fear. In the meanwhile, the tube is being rapidly propelled down the esophagus, and generally a little sound of escaping gas can be detected as the tube slips through the cardiac orifice.

Ordinarily a distance of twenty or twenty-one inches will put the tube sufficiently into the stomach, but there are many variations to this rule. Tall patients, or those with prolapsed stomachs will require more inches, while I have had diminutive patients whose stomach cavities were little over 18 inches from the teeth.

I well remember one tall and slender old lady, with relaxed abdominal parietes and a stomach ptosed practically into her pelvis, who required nearly thirty inches of tube in order to either aspirate or irrigate her stomach.

At this time the patient should sit erectly, with the head slightly inclined forward, as this position minimizes the nausea. This may require some reminding or gentle urging, for nearly every one is inclined to rear backward, with the head thrown still further backward, and the mouth wide open, all of which augments instead of decreasing the discomfort.

It is well at the very first to introduce the tube just a little father than is necessary, so as to permit of gradually drawing it out during the aspiration, should the end of the tube not engage itself in the stomach contents at once.

As to the methods of aspirating the contents, they, too, are legion.

The "expression method" consists of directing the patient to place his hands over his stomach, and with both external pressure and abdominal contraction, as in the effort to empty the intestines, the contents of the stomach may be forced out of the tube. This is a good method when it succeeds, only it rarely succeeds, and it entails too much effort on the patient's part.

Others use a large Politzer bag, aspirating the contents into this bag. The objection to this lies in the fact that the operator cannot know how much of the contents he has obtained, and also, if he is not careful, he is liable as he squeezes the bag, to force the contents back into the stomach as fast as he aspirates them out.

By far the best method in my hands is the simple, valveless bulb, known as the Lockwood bulb, and furnished by Tiemann & Co., of New York. This is made of good rubber, is highly resillient, and, possessing no valves, either end can be inserted into the end of the tube. In aspirating the stomach contents, the bulb is compressed and one of the operator's fingers is placed over the open end, acting as a valve, and the pressure being quickly removed from the bulb, it expands and ressumes its form, creating a vacuum, and drawing up the contents of the stomach. The finger is then removed from the end, and the next pressure empties the contents of the bulb into the waiting receptacle. This can be rapidly repeated, and any operator can soon get the "knack." Should the tube seem clogged, by first placing the finger over the open end, and then quickly compressing the bulb, air is forced through the tube, and the obstruction easily cleared.

Should the efforts at aspiration fail to bring anything, it is well to gently and slowly draw forward the tube, while these efforts are kept up, as sometimes the end of the tube is not in the fluid contents at the most dependent part of the stomach. Should it be drawn on out until, by the easy escape of air, the operator knows that the tube has almost left the stomach, it may be pressed downward again, and the same manipulation repeated. This may be done several times until either the efforts are successful or the operator is convinced that the stomach is empty.

Occasionally in stomachs of irregular conformation, the contents are extremely hard to locate, and it will require considerable patience on the part of the physician to guide the end of the tube into the small space where the tube may be engaged therein. Intelligent efforts, however, assisted by forbearance and co-operation on the part of the patient, will nearly always result in success.

In cases of very patulous pylorus, or hypermotility of the stomach, the viscus may be absolutely empty, and of course efforts at aspiration will be futile.

It is seldom necessary to attempt to thoroughly evacuate the stomach when getting a test meal. From fifteen to twenty-five cubic centimeters are sufficient for any ordinary quantitatives tests, and it is not wise to needlessly prolong the operation. Under favorable circumstances from two to four expansions of the bulb will aspirate this, and then the tube should be rapidly drawn from the stomach with one gentle but firm pull. The two fingers of the operator that grasp the tube should also compress it, so that no random drops of fluid may run out on the floor or clothing of the patient as it emerges from the mouth. This little precaution is worth noting.

Though considerable time has been taken in describing these various manipulations, the act of introducing a tube into a patient's stomach, aspirating a test meal, and withdrawing the tube, should require but a few seconds. I have many times completed the whole act in from four to six seconds, and, unless some hindrance intervenes, it should nearly always be completed inside of thirty seconds. If the operator finds that it is taking him longer than this, he should endeavor to hasten the different steps, so as to come within this time limit.

There is no procedure in the whole range of therapeutics wherein deftness and celerity on the part of the physician is more appreciated than in the introduction of the stomach tube. Let me, therefore, urge my readers to painstakingly practice this really simple operation, so that the people at large may lose that fear and horror of the stomach tube, and may, instead of looking upon it as an instrument of turture, accord it the place it deserves, as a most useful adjunct in the diagnosis and treatment of diseases of the alimentary tract.

